# The role of *MXRA7* in bone marrow senescence involves macrophage polarization and microenvironment remodeling

**DOI:** 10.1097/BS9.0000000000000287

**Published:** 2026-05-28

**Authors:** Yuzhen Qin, Ziyan Zhao, Yihan Chen, Yudan Zheng, Kunpeng Ma, Dandan Lin, Xin Liu, Yiqiang Wang

**Affiliations:** a Wisdom Lake Academy of Pharmacy, Xi’an Jiaotong-Liverpool University, Suzhou 215123, China; b MOH Key Lab of Thrombosis and Hemostasis, Jiangsu Institute of Hematology, The First Affiliated Hospital of Soochow University, Soochow University, Suzhou 215006, China; c Central Lab, Xiamen University Medical Center, Xiamen University, Xiamen 361005, China

**Keywords:** Aging, Bone marrow microenvironment, Macrophage polarization, *MXRA7*, scRNA-seq

## Abstract

Marrow senescence contributes to overall organismal aging and involves functional alterations in both the mesenchymal and hematopoietic compartments of bone marrow. Although matrix remodeling-associated 7 (*MXRA7*) has been demonstrated to modulate mesenchymal function and megakaryocyte differentiation in mice, this study aimed to investigate the potential role of *MXRA7* in marrow senescence. Single-cell RNA sequencing was performed on bone marrow cells from young and aged wild-type and *MXRA7*-knockout mice. Comparative analysis of 2-month-old and 2-year-old mice revealed that aging significantly altered the cellular proportions within the bone marrow niche, and *MXRA7* deficiency markedly increased Macro1 macrophages in aged mice, likely driven by the dysregulation of the *Ccl24-Ccr3* axis. *MXRA7* deficiency altered Mid1 expression and the macrophage migration inhibitory factor (*Cd74 + Cxcr4*), *Ccl6 + Ccr2*, and Von Willebrand factor signaling pairs (*Itga2b + Itgb3*), all of which are closely associated with cell status in the bone marrow microenvironment. In summary, these findings underscore *MXRA7*’s role in cellular profile shifts during bone marrow aging, offering novel insights into how *MXRA7* coordinates hematopoietic and immune homeostasis in the bone marrow.

## 1. INTRODUCTION

Organismal aging represents the cumulative outcome of the progressive, programmed dysfunction across multiple organs/systems toward the end of life. Among body-forming systems, 3 that involve the bone marrow (ie, the skeletal, immune, and blood-vascular systems) play important roles in determining the overall physiological status, including susceptibility to and pathogenesis of aging-associated diseases, such as metabolic, degenerative, or carcinogenic disorders.^1,2^ Thus, an improved understanding of bone marrow senescence will facilitate better understanding and management of aging. Similar to any other highly dynamic biological process, hematopoiesis and osteogenesis rely on extracellular matrix remodeling,^3^ which is determined by the net results of interactions among all soluble, particulate, and cellular components in the bone marrow compartments.

Conversely, at the core of life, all biological activities are determined by the gene products. However, only a small fraction of gene-encoded information traits is currently known, rendering current biomedical theories and practices based on this “known” genomic information (ie, “knome”) incomplete or potentially inaccurate.^4^ To better understand health- or disease-related issues, exploring the unknown kingdom or “genomic territory” is both vast and urgent. Inspired by this vision, the Wang team has investigated the biological functions of the gene matrix remodeling-associated 7 (*MXRA7*) across different tissues and conditions.^5–11^ Concerning bone marrow or blood science alone, *MXRA7* was identified to be involved in the regulation of mesenchymal stem cell differentiation toward osteoblasts or adipocytes,^8^ megakaryocyte differentiation,^10^ acute promyelocytic leukemia cell differentiation,^9^ and monocyte polarization toward Macro1 (M1) macrophages.^11^ Based on this rationale, we hypothesized that *MXRA7* may also play a role in bone marrow senescence. To test this hypothesis, single-cell RNA sequencing (scRNA-seq) was performed on bone marrow cells from 2-month-old (young) and 2-year-old (aged) mice with either wild-type (WT) *MXRA7* expression or *MXRA7*-deficient (*MXRA7*-KO) expression. This study offers a preliminary analysis of the dataset, revealing the significant impact of *MXRA7* deficiency on macrophage polarization during senescence in the bone marrow microenvironment of mice.

## 2. MATERIALS AND METHODS

### 2.1. Animals

Animal experiments were approved by the Institutional Laboratory Animal Care and Use Committee of Soochow University (No.2016-075-1) following the Guidelines on the Humane Treatment of Laboratory Animals (Ministry of Science and Technology of China, 2006). Heterozygous (*Mxra7*^+/−^) breeders on C57BL/6N background were obtained from Medical Research Council (MRC, Swindon, UK) and maintained in a specific pathogen-free facility of Soochow University to produce the WT and KO mice via crossbreeding as described earlier.^8^

### 2.2. Cell sample preparation and scRNA sequencing

Seven- to 8-week-old (2 months or 2m) or 25-month-old (2 years or 2Y) WT and KO mice (ie, WT2m, X2m, WT2Y, X2Y), 5 per group, were euthanized, and 1 femur from each mouse was used for single-cell preparation as routinely performed. Cells from 5 mice in each group were pooled, respectively, passed through 40-μm nylon sterile filters, and erythrocytes were removed via the ammonia chloride lysis method. Viability of cells in the final cell suspensions was assessed using Countess^®^ II Automated Cell Counter (Thermo Fisher Scientific, Waltham, Massachusetts) and determined to exceed 90%. Single-cell suspension was subjected to Chromium Single Cell 3′ Reagent v2 kits (10× Genomics) to generate barcoded scRNA-seq libraries in accordance with the manufacturer’s protocol, the details of which and subsequent experimental procedures are beyond the scope of this article.

### 2.3. Data processing and quality control

Raw sequencing data were collected and quality-controlled using Illumina’s data collection software, and further processed using Cell Ranger software (version 2.0.1, 10× Genomics). After sequential demultiplexing of cellular barcodes, mapping of reads to the genome and transcriptome, and downsampling of reads as necessary, normalized aggregate data across samples were obtained. Data quality control and filtering were performed by importing CellRanger-generated data into Seurat objects using the R packages Seurat (v5.1.0)^12^ and RStudio (v4.4.2). Cells expressing fewer than 3 genes or containing fewer than 200 detected genes were retained, while those with <500 or >4000 detected genes or mitochondrial gene content exceeding 10% were filtered to remove potential doublets. Following normalization of unique molecular identifier counts using the LogNormalize method, the top 2000 highly variable genes per sample were identified based on mean expression and dispersion (variance-to-mean ratio) for integration. The samples were merged using the merge function, and batch effects were corrected using Harmony.^13^ Dimensionality reduction was performed using principal component analysis (30 principal components), followed by uniform manifold approximation and projection. Clustering using the FindClusters function (default resolution: 0.5) generated 24 initial clusters, which were annotated to 10 major cell types by cross-referencing the CellMarker 2.0 database,^14^ and the hematopoietic subpopulations were further annotated based on the ABC portal database.^15^ A heat map was generated using the scRNAtoolVis package (https://github.com/junjunlab/scRNAtoolVis). Additionally, a volcano plot of the differential gene expression was generated using EnhancedVolcano (https://github.com/kevinblighe/EnhancedVolcano). Gene Ontology (GO) and Kyoto Encyclopedia of Genes and Genomes (KEGG) pathway analyses were subsequently performed using the clusterProfiler package and Metascape database^16^ to identify the biological functions and pathways enriched in the differentially expressed genes. Monocle2 was employed for pseudotime analysis (https://bioconductor.org/packages/monocle), and CellChatV2 was used for cell–cell communication analysis.^17^ To strengthen the rigor of cell proportion analysis, we performed statistical comparisons of cell subpopulations across different samples using the prop.test function in R. More details of data analysis are provided in SDC, Analysis Methods (https://links.lww.com/BS/A154).

### 2.4. Data availability

Raw sequencing data files were uploaded to the National Center for Biotechnology Information under the Gene Expression Omnibus accession number GSE255023. The codes used in this study can be retrieved from https://github.com/YihanCell/Mxra7-2M2Y. Additional information supporting the data analysis reported in this study is available upon request.

## 3. RESULTS AND DISCUSSION

### 3.1. *MXRA7* deficiency altered the cellular and transcriptomic profile change patterns of bone marrow in aged mice

**Figure 1.**
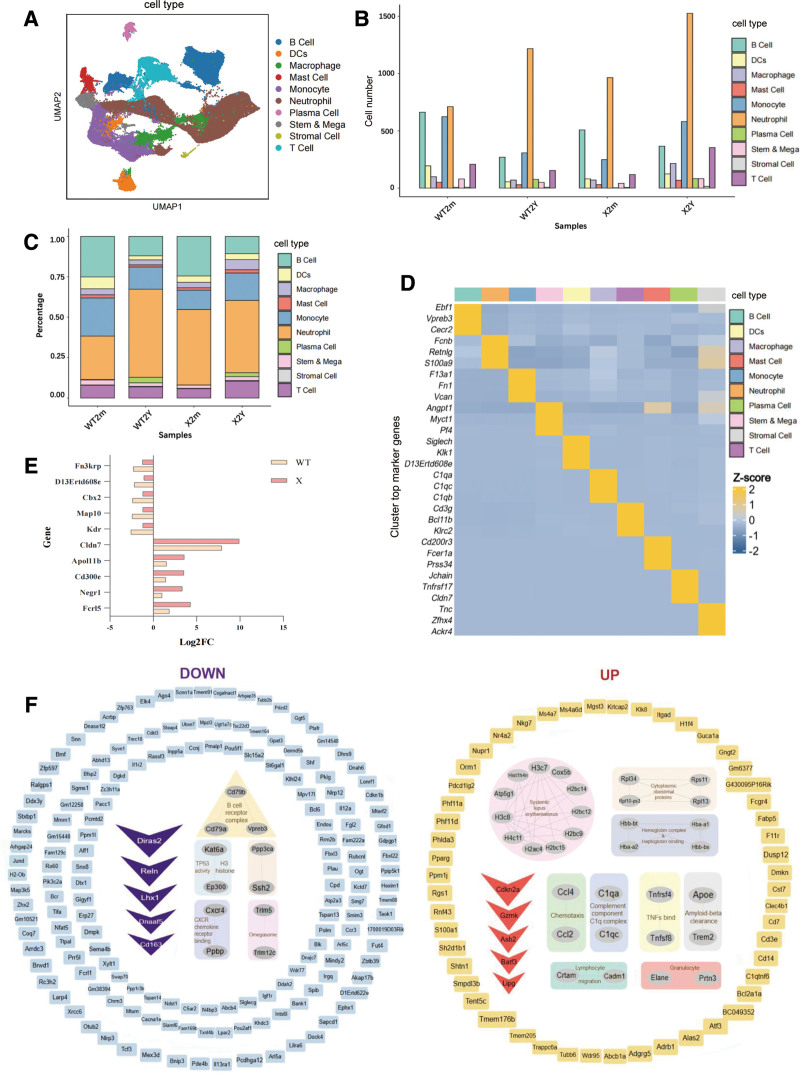
Changes in bone marrow cell atlas under matrix remodeling-associated 7 (*MXRA7*) deficiency. (A) Uniform manifold approximation and projection of total samples and cell type, clustering with the FindClusters function (resolution: 0.5), which were annotated to 10 major cell types by the CellMarker 2.0 database (http://bio-bigdata.hrbmu.edu.cn/CellMarker). (B–C) The proportion and number distribution of cell types in different samples. (D) Heatmap of cell type–specific highly expressed genes conducted by scRNAtoolVis (https://github.com/junjunlab/scRNAtoolVis). (E) Bar chart of fold changes in commonly differentially expressed genes (top 5 upregulated and downregulated genes) between wild-type (meat-colored) and *MXRA7*-deficient (pink) mice due to aging. The x-axis represents the fold change (Log2FC), and the y-axis lists the gene names. Created using GraphPad Prism 8. (F) Unique gene expression changes associated with aging in *MXRA7*-deficient mice. Downregulated genes are displayed on the left, with the top 5 downregulated genes in dark purple and other downregulated genes in light blue. Upregulated genes are displayed on the right, with the top 5 upregulated genes in red and other upregulated genes in orange. Functional clustering was performed using the STRING database, and the visualization was created using Cytoscape v3.10.1.

After a smooth workflow (SDC, Workflow, https://links.lww.com/BS/A154), a total of 121,785 cells, including 31,164 cells from WT2m, 25,807 cells from WT2Y, 25,977 cells from X2m, and 38,837 cells from X2Y, respectively, passed validation and normalization, and were included for further population analysis based on gene expression. Clustering across all 4 groups yielded 24 cell clusters after excluding mature red blood cell contaminants (SDC Figure S1C, https://links.lww.com/BS/A154). Following annotation with recognized marker genes (SDC Figure S1B, https://links.lww.com/BS/A154),^14^ 10 distinct cell types were identified (**Fig. 1A**), and cell populations are displayed separately for each sample (SDC Figure S1A, https://links.lww.com/BS/A154). As expected, the composition of all cells (**Fig. 1B** and **C**, detailed later) and summative gene expression (**Fig. 1D**) were altered by aging and *MXRA7* deficiency. A comparison of the transcriptomic profiles revealed 1824 differentially expressed genes among 10,174 genes detected in WT mice, including 221 upregulated and 1603 downregulated genes in aged vs young mice. Similarly, 178 genes were upregulated, and 613 were downregulated in the X2Y/X2m comparison (SDC Figure S2A, https://links.lww.com/BS/A154). GO enrichment analysis demonstrated that genes upregulated during WT aging were mainly involved in peptide cross-linking, epidermal cell differentiation, and cell–cell adhesion, whereas downregulated genes were associated with deoxyribonucleic acid (DNA) damage response, DNA repair, and chromosomal organization (SDC Figure S2B, https://links.lww.com/BS/A154). KEGG analysis further indicated that genes upregulated during WT aging were associated with pathways such as *Salmonella* infection, non-alcoholic liver disease, osteoclast differentiation, prolactin signaling, and cytokine–cytokine receptor interaction, whereas downregulated genes were enriched in the cell cycle, cellular senescence, human T-cell leukemia virus type 1 infection, nucleocytoplasmic transport, and Fanconi anemia pathways (SDC Figure S2B, https://links.lww.com/BS/A154). In contrast, *MXRA7*-KO mice exhibited distinct transcriptome changes on aging. GO enrichment analysis highlighted the upregulated genes involved in leukocyte migration, myeloid cell differentiation, immune response, and inflammatory regulation, whereas the downregulated genes were strongly associated with immune cell differentiation and cell adhesion (SDC Figure S2B, https://links.lww.com/BS/A154). KEGG analysis also revealed that genes upregulated during *MXRA7*-KO aging were enriched in pathways such as systemic lupus erythematosus, neutrophil extracellular traps, alcoholism, viral carcinogenesis, malaria, and hematopoietic cell lineage, whereas downregulated genes were associated with B-cell receptor signaling, transcriptional misregulation in cancer, osteoclast differentiation, prostate cancer, and the human immunodeficiency virus type 1 viral life cycle (SDC Figure S2B, https://links.lww.com/BS/A154). Transcriptomic alterations in WT aging primarily manifest as impaired cellular integrity and diminished DNA function, whereas aging in *MXRA7*-KO mice leads to the widespread activation of immune and inflammatory pathways. This indicates that *MXRA7* deficiency alters the core molecular phenotype of aging, steering it from a functional decline in maintenance processes toward prominent immune dysregulation.

As illustrated in **Figure 1F**, compared to X2m mice, X2Y mice exhibited significant upregulation of *Cdkn2a*, *Gzmk*, *Asb2*, *Batf3*, and *Lipg*, alongside downregulation of *Diras2*, *Reln*, *Lhx1*, and *Dnaaf5*. Furthermore, a comparative analysis of shared differentially expressed genes between WT and *MXRA7*-KO mice (**Fig. 1E**) revealed more pronounced dysregulation in *MXRA7*-KO mice; upregulated genes (*Fcrl5*, *Negr1*, *Cd300e*, *Apol11b*, and *Cldn7*) and downregulated genes (*Kdr*, *Map10*, *Cbx2*, *D13Ertd608e*, and *Fn3krp*) demonstrated greater changes compared to those in WT mice. A summary of these genes is presented in Table 1.

**Table 1:** Different-expressed genes in aging.

Change pattern	Gene symbol	Function and mechanism	Related diseases/models
Unique Changes in *MXRA7*-KO			
Upregulated	*CDKN2A*	Tumor suppressor, marker of cellular senescence	AD models, aging studies
	*GZMK*	Cytotoxic T-cell marker, promotes inflammation; associated with immunosenescence, infections, and cancer	COVID-19, AML, burn injury models
	*ASB2*	Subunit of E3 ubiquitin ligase, regulates hematopoiesis and myogenesis	Leukemia, muscle atrophy
	*BATF3*	Regulates cDC1 development, enhances anti-tumor and anti-pathogen immunity	Listeria infection models, cancer immunity
	*LIPG*	Endothelial lipase regulates lipid metabolism and inflammation; promotes atherosclerosis, AML, and breast cancer	Atherosclerosis, AML, triple-negative breast cancer
Downregulated	*DIRAS2*	Regulates macrophage infiltration/polarization, suppresses CCL2-mediated TME remodeling	Triple-negative breast cancer
	*RELN*	Extracellular glycoprotein, inhibits α-synuclein aggregation, protects neuronal survival	Parkinson disease, brain aging
	*CD163*	M2 macrophage marker, regulates anti-inflammation and tissue repair; promotes tumor progression	Neurodegeneration, atherosclerosis, cancer
Shared changes by WT and *MXRA7*-KO			
Upregulated	*FCRL5*	IgG receptor, enhances B-cell activity; overexpression triggers autoimmunity	Systemic lupus erythematosus
	*NEGR1*	Neuronal growth regulator, suppresses M2 macrophage polarization	Colorectal cancer liver metastasis
	*CD300e*	Pro-inflammatory receptor, regulates monocyte differentiation; linked to MDS prognosis and COVID-19 severity	MDS, OA, COVID-19
	*CLDN7*	Tight junction protein, maintains renal epithelial barrier; linked to ischemic kidney injury	Renal aging, ischemic nephropathy
Downregulated	*KDR (VEGFR-2*)	Angiogenesis regulator, supports HSC survival; dysregulated in cancer and leukemia	Solid tumors, leukemia
	*CBX2*	Polycomb protein, regulates hematopoiesis and development; promotes leukemia progression	AML, sex determination disorders
	*FN3KRP*	Reverse glycation enzyme, longevity-associated; rs1046896-C allele promotes longevity	Aging and longevity studies

AD = Alzheimer’s disease, AML = acute myeloid leukemia, CCL2 = chemokine (C-C motif) ligand 2, COVID-19 = coronavirus disease 2019, MDS = myelodysplastic syndromes, OA = osteoarthritis.

### 3.2. *MXRA7* deficiency altered macrophage M1 polarization in aged mice

**Figure 2.**
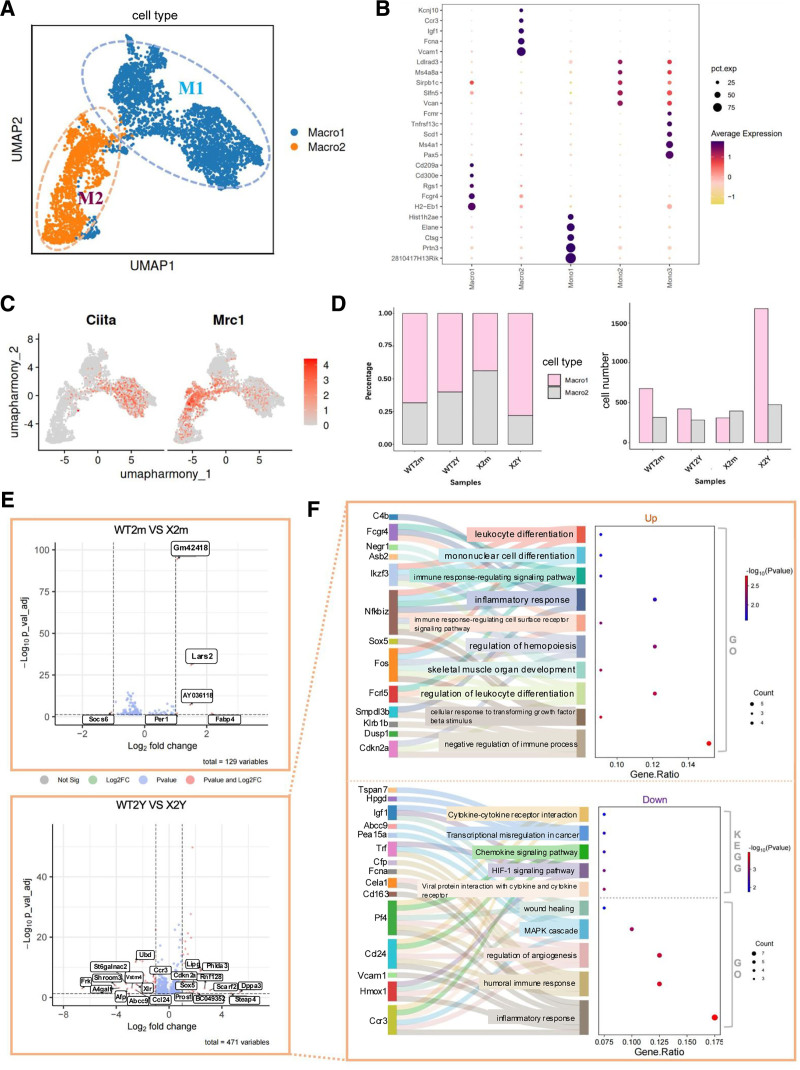
Gene expression changes in macrophage subtypes under matrix remodeling-associated 7 (*MXRA7*) deficiency. (A) Macrophage clustering into M1 and M2 subtypes based on canonical markers. (B) Dot plot of subtype-specific highly expressed genes in monocytes and macrophages. (C) Marker gene of M1 (*Ciita*) and M2 (*Mrc1*) macrophages. (D) M1/M2 macrophage proportions: young *MXRA7*-KO mice exhibited an initial increase in M2 macrophages, whereas aged *MXRA7*-KO mice demonstrated predominant M1 expansion. (E–F) Enrichment analysis of age-dependent differentially expressed genes in *MXRA7*-KO mice, revealing co-downregulation of *Ccl24* and in aged cohorts; the volcano plot was generated using EnhancedVolcano (https://github.com/kevinblighe/EnhancedVolcano), and visualized via http://www.bioinformatics.com.cn.

Cellular composition analysis of the 4 samples demonstrated that WT2Y mice had more neutrophils and plasma cells but fewer dendritic cells, monocytes, and B cells (**Fig. 1C**; SDC Figure S3C, https://links.lww.com/BS/A154). X2m mice exhibited an aging-like profile, with increased neutrophils and reduced monocytes and dendritic cells. However, X2Y mice had more macrophages and T cells with fewer neutrophils than those in WT2Y mice (**Fig. 1C**, SDC Figure S3C, https://links.lww.com/BS/A154). Given that macrophages are known to contribute to bone marrow senescence,^2^ they were the focus of subsequent analyses. Macrophages are routinely classified into 2 subsets: M1 and Macro2 (M2) (**Fig. 2A–C**).^18–22^ The current study revealed apronounced effect of *MXRA7*-KO on M1 macrophages in aged mice (**Fig. 2D**). A volcano plot of X2Y/WT2Y comparison (**Fig. 2E**) based on gene expression revealed that the differentially expressed genes were predominantly associated with immune response, inflammation, cell differentiation, and cytokines, but with significant differences between up- and downregulated genes (**Fig. 2F**). *MXRA7* deficiency reprogrammed the gene expression profile of aged macrophages, shifting them from a homeostatic-responsive state to a hyperactive, dysregulated immune-activated state.

Further sub-clustering of the monocyte-macrophage population revealed 3 distinct subsets (SDC Figure S3A, https://links.lww.com/BS/A154). Developmental trajectory analysis of monocytes and macrophages was performed using Monocle2 software (SDC Figure S3B, https://links.lww.com/BS/A154), which identified 3 pseudotime states. Visualization of cell types across these states (SDC Figure S3C, https://links.lww.com/BS/A154, right) demonstrated that monocyte subset Mono1 dominated the early differentiation phase, whereas Mono2 and Mono3 were more prevalent in the mid-to-late phases. Among the macrophages, M1 maintained a high proportion across all stages, whereas M2 emerged predominantly in the late pseudotime, particularly in Fate1 and Fate3. Notably, the sample analysis (SDC Figure S3C, https://links.lww.com/BS/A154, left) exhibited a markedly increased proportion of M1 macrophages in X2Y mice. Subsequent analysis of genes associated with monocyte-macrophage trajectories (SDC Figure S3D, https://links.lww.com/BS/A154, left) categorized these into 3 clusters based on the pseudotime progression: early, middle, and late. Functional enrichment analysis of these clusters was conducted to elucidate their biological roles, with a focus on Cluster3 (late-stage genes). In the late developmental stages, monocytes and macrophages shift from proliferation and differentiation toward functional specialization. This includes matrix remodeling, signal modulation, initiation of inflammatory responses, and metabolic adaptation, thereby preparing them for terminal roles, such as immune surveillance, tissue repair (M2), and regulation of inflammation.

### 3.3. *MXRA7* deficiency altered the overall bone marrow microenvironment

**Figure 3.**
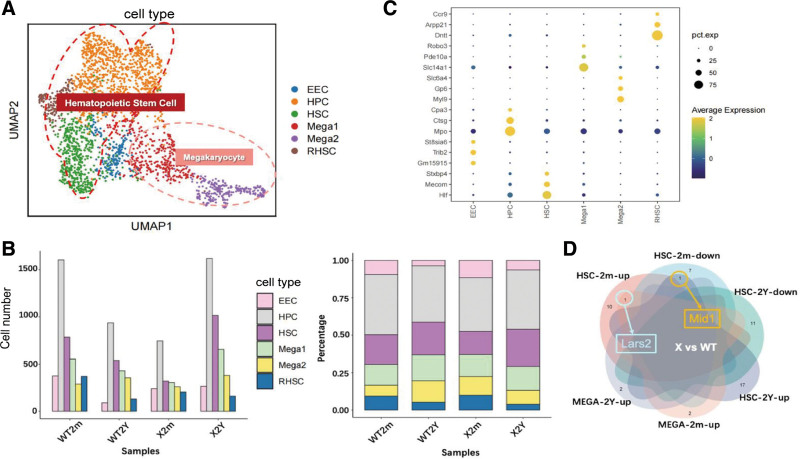
Gene expression changes in HSCs and megakaryocytes under matrix remodeling-associated 7 (*MXRA7*) deficiency. (A) Classification of hematopoietic populations: early erythroid cells, hematopoietic progenitor cells, HSCs, resident HSCs, and 2 megakaryocyte subsets. (B) Cell numbers and proportions across hematopoietic clusters. (C) Dot plot of subpopulation-specific highly expressed genes. (D) Venn diagram of differentially expressed genes in HSCs and megakaryocytes under *MXRA7* deficiency; *Mid1* is downregulated in HSCs of young mice but upregulated in aged mice, while *Lars2* is upregulated in both HSCs and megakaryocytes of young mice. The plot was created by http://www.bioinformatics.com.cn. HSCs = hematopoietic stem cells.

Self-renewal, proliferation, and differentiation of hematopoietic stem cells (HSCs) are governed by a highly sophisticated regulatory network known as the HSC microenvironment or niche. Within this niche, HSC fate decisions are dynamically modulated by supportive cells and signaling cues.^23^ The HSC niche includes mesenchymal stem cells, megakaryocytes, osteoblasts, osteoclasts, macrophages, neutrophils, and adipocytes, which collectively maintain or regulate hematopoietic homeostasis.^24,25^ Previous studies have established that *MXRA7* regulates bone marrow mesenchymal stem cell differentiation toward osteoblast or adipocyte,^8^ as well as megakaryocyte differentiation. Differential changes in macrophages and neutrophils in aged *MXRA7*-KO and WT mice (**Fig. 3B**) demonstrated that nearly all dominant cell populations, except blast cells, exhibited age- and *MXRA7*-dependent alterations. To elucidate the regulatory dynamics of HSCs and megakaryocytes, we performed in-depth subpopulation analysis (**Fig. 3A**). The original 8 clusters and their corresponding marker genes for each cell type were also plotted (SDC Figure S4A and B, https://links.lww.com/BS/A154). Highly expressed subtype-specific genes are illustrated in **Figure 3C**. Subsequent quantification of cell numbers and proportions across samples (**Fig. 3B**) revealed that young *MXRA7*-KO mice exhibited a significant reduction in all hematopoietic cell populations, particularly hematopoietic progenitor cells (HPCs). Consistent with prior findings, *MXRA7*-KO mice displayed lower megakaryocyte counts in both the bone marrow and spleen than the counts observed in the WT group.^10^ Under *MXRA7* deficiency, we also observed alterations in the expression of *Mid1* and *Lars2* (**Fig. 3D**). Notably, while aging in WT mice caused a decline in most hematopoietic cell types, *MXRA7*-KO mice exhibited partial recovery in old age following severe reductions in youth, with HPCs demonstrating the most pronounced rebound (**Fig. 3B**). Our re-analysis of the public dataset (GSE11504) revealed that the expression of *MXRA7* during normal bone marrow development exhibited a dynamic pattern of initial decrease, followed by a subsequent increase from infancy to the young adulthood (SDC Figure S4D, https://links.lww.com/BS/A154). Although this change did not reach statistical significance, likely due to the absence of an older group in the analysis, these results suggest that the expression of *MXRA7* may be finely regulated by age or developmental stage.

### 3.4. Cell–cell interactions, which change with aging, are influenced by *MXRA7* deficiency

To explore how *MXRA7* affects cellular communication within the bone marrow microenvironment, CellChat was utilized to analyze ligand–receptor interactions among various cell populations in WT and *MXRA7*-KO samples (SDC Figure S5A, https://links.lww.com/BS/A154), with detailed ligand–receptor interactions demonstrated for HSCs (SDC Figure S5B, https://links.lww.com/BS/A154) and megakaryocytes (SDC Figure S5C, https://links.lww.com/BS/A154) across all samples. The analysis revealed that the macrophage migration inhibitory factor (Mif) ligand–receptor pairs were predominant. Specifically, the loss of the Mif (*Cd74+Cxcr4*) pair between HSCs/megakaryocytes and macrophages in aged *MXRA7*-KO mice was observed. Additionally, *MXRA7* deficiency eliminated the *Ccl6+Ccr2* pair between HSCs/megakaryocytes and macrophages, as well as the Vwf (*Itga2b+Itgb3*) interaction in HSCs/megakaryocytes of aged *MXRA7*-KO mice (SDC Figure S5B and C, https://links.lww.com/BS/A154).

In summary, integration of scRNA-Seq data from cells across young and aged WT and *MXRA7*-KO cohorts revealed landscape alterations in the bone marrow senescence, with *MXRA7* deficiency causing age-specific microenvironmental imbalance, especially in aged KO mice (X2Y) exhibiting macrophage expansion with M1-subtype dominance in the bone marrow. This observation aligns with findings that macrophage polarization is responsible for hematopoietic regulation under either physiological or pathological conditions.^26–28^ We noted that the expression of *MXRA7* was significantly decreased during bone marrow aging in WT mice (SDC Figure S4E, https://links.lww.com/BS/A154). This trend was further supported by RNA-seq data from a public dataset (GSE243327), which demonstrated *MXRA7* downregulation in aged human hematopoietic stem/progenitor cells (SDC Figure S4F, https://links.lww.com/BS/A154). Interestingly, analysis of a mouse multi-organ aging dataset (GSE247440) revealed that *MXRA7* expression was upregulated in aged livers and kidneys. In a study by Hong et al,^29^ *MXRA7* was identified as one of the genes whose expression significantly increased with age in lymphocytes. At the molecular level, differentially expressed genes across genotypes and ages (**Fig. 1F**) suggested that *MXRA7* deficiency disrupts bone marrow homeostasis via synergistic epigenetic, metabolic, and inflammatory pathways. For instance, *ASB2* dysregulation impedes the ubiquitin-dependent degradation of filamin proteins and directly blocks hematopoietic differentiation.^30^ *CBX2* suppression destabilizes polycomb-mediated epigenetic regulation, inducing a HSC differentiation bias and leukemic transformation.^31^ Metabolically, *LIPG* overexpression exacerbates lipid inflammatory responses through lysophosphatidylcholine generation, promoting macrophage lipid accumulation, and supporting leukemic cell survival via lipid droplet formation.^32–34^ Regarding niche maintenance, *KDR* downregulation compromises angiogenesis and hematopoietic niche function.^35^ Similarly, altered intercellular communication is a hallmark of aging.^36^ In the aged *MXRA7*-KO mice, the present study demonstrated that *MXRA7* deficiency disrupts the Mif (*Cd74 + Cxcr4*) and *Ccl6 + Ccr2* ligand–receptor pairs, which may promote M1 macrophage polarization via the nuclear factor kappa-light-chain-enhancer of activated B cells (NF-κB) and PI3K/Akt pathways. Concurrently, impaired Vwf (*Itga2b + Itgb3*) ligand–receptor function through NF-κB signaling suppressed HSCs and megakaryocyte activities. Therefore, the dynamic changes of *MXRA7* during aging, especially across species and multiple organ systems, require further in-depth investigation. In future studies, employing hematopoietic system‑specific *MXRA7*‑KO mice will help refine the experimental approach and precisely elucidate the role of *MXRA7* in bone marrow hematopoiesis and aging.

## 4. CONCLUSION

Although bone marrow aging involves intricate interactions among niche cells, including mesenchymal cells, megakaryocytes, and macrophages, the current study highlights that *MXRA7* deficiency drives increased M1 macrophage polarization during aging through multidimensional regulatory networks and intercellular crosstalk. Given that this team had demonstrated that *MXRA7* regulates all three main niche cell types, we proposed that *MXRA7* plays important roles in bone marrow senescence and may even serve as a novel target for diseases associated with bone marrow biology.

## ACKNOWLEDGMENTS

This work was supported by the National Natural Science Foundation of China (81600076, 81271050), and partially supported by Xiang’an Hospital of Xiamen University (PM20205180001 to Y.W.) and the SIP High-quality Innovation Platform for Chronic Diseases at the Wisdom Lake Academy of Pharmacy, XJTLU, Suzhou, China.

We acknowledge the CellMarker database, Metascape database, ABC portal, and the Biomamba team for their resources and support. We also thank the Biomamba (Nanjing, Jiangsu, China) team for insightful discussion.

## AUTHOR CONTRIBUTIONS

D.L. and Y.W. participated in conceptualization and funding acquisition; Y.Q., Y.C., X.L., and Y.W. participated in methodology; Y.Q. and Y.C. participated in formal analysis; Y.W. participated in resources, writing—review and editing, and project administration; Y.Q. Z.Z., and Y.C. participated in data curation; Y.Q. and Z.Z. participated in writing—original draft preparation; Y.Q. participated in visualization; Y.W. and X.L. participated in supervision. All authors have read and agreed to the submitted version of the manuscript.

## Supplementary Material
