# Supplementary material for: The role of MXRA7 in bone marrow senescence involves macrophage polarization and microenvironment remodeling
Source: Blood Sci. 2026 May 28;8(2):e00287. doi: 10.1097/BS9.0000000000000287 (PMC13221116; doi:10.1097/BS9.0000000000000287)
Supplement: Supplementary file 1 [file bs9-8-e00287-s001.pdf]

SUPPLEMENTARY

The role of MXRA7 in bone marrow senescence involves macrophage polarization and microenvironment remodeling

Yuzhen Qin[a], Ziyan Zhao[a], Yihan Chen[a], Yudan Zheng[b], Kunpeng Ma[b], Dandan Lin[b] , Xin Liu[a], Yiqiang Wang[a,b,c *]

[a]Wisdom Lake Academy of Pharmacy, Xi'an Jiaotong-Liverpool University, Suzhou 215123, China;
[b]MOH Key Lab of Thrombosis and Hemostasis, Jiangsu Institute of Hematology, The First Affiliated Hospital of Soochow University, Soochow University, Suzhou 215006, China;

[c]Central Lab, Xiamen University Medical Center, Xiamen University, Xiamen 361005, China

SDC, Workflow：

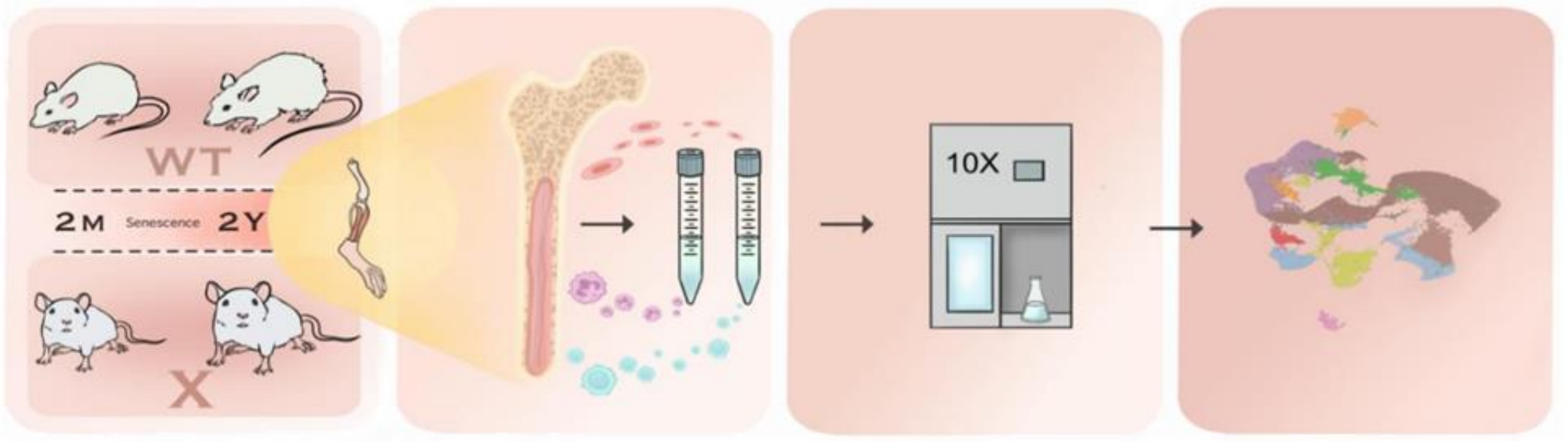

Animal preparation **Single cell sampling** **scRNA-sequencing** **Data analysis**

SDC, Analysis Methods：

All bioinformatics analyses were performed in the R language environment (v4.4.2) using the R Studio development environment. Single-cell data processing and analysis were primarily based on the Seurat platform (v5.1.0). Briefly, the count matrices generated by CellRanger were imported into Seurat objects for quality control and filtering. Cells with the number of expressed genes between 200 and 4,000 and mitochondrial gene content below 10% were retained to remove low-quality cells. The data were normalized using the "LogNormalize" method, and the top 2,000 highly variable genes per sample were identified based on the variance stabilizing transformation (vst) algorithm. Subsequently, all samples were merged using the Merge function, and batch effects were corrected using the Harmony (v1.2.0).

Dimensionality reduction and clustering analyses were performed based on the top 30 principal components (PCA) and visualized using Uniform Manifold Approximation and Projection (UMAP). Unsupervised clustering was conducted using the FindClusters function (resolution set to 0.5), which identified a total of 24 initial cell clusters. Cell type annotation was accomplished by cross-referencing the CellMarker 2.0 database, defining 10 major cell types. Hematopoietic-related subpopulations were further annotated based on the ABC portal database.

Differential expression analysis was performed using the FindMarkers function in Seurat, and the results were visualized as volcano plots using the EnhancedVolcano package. Functional enrichment analysis was conducted using the clusterProfiler suite (v4.13.1) combined with the org.Mm.eg.db annotation database (v3.20.0) within the framework of the Metascape database, performing Gene Ontology (GO) and Kyoto Encyclopedia of Genes and Genomes (KEGG) pathway enrichment analyses. Pseudotime analysis was performed using Monocle2. Cell-cell communication network analysis was inferred and visualized using CellChatV2. To enhance the rigor of the cell proportion analysis, statistical comparisons of cell subpopulation proportions between different samples were performed using the prop.test function from the R base package. All statistical plots were generated and arranged using ggplot2 (v3.5.1) in combination with packages such as patchwork (v1.2.0), paletteer (v1.6.0), and scRNAtoolVis.

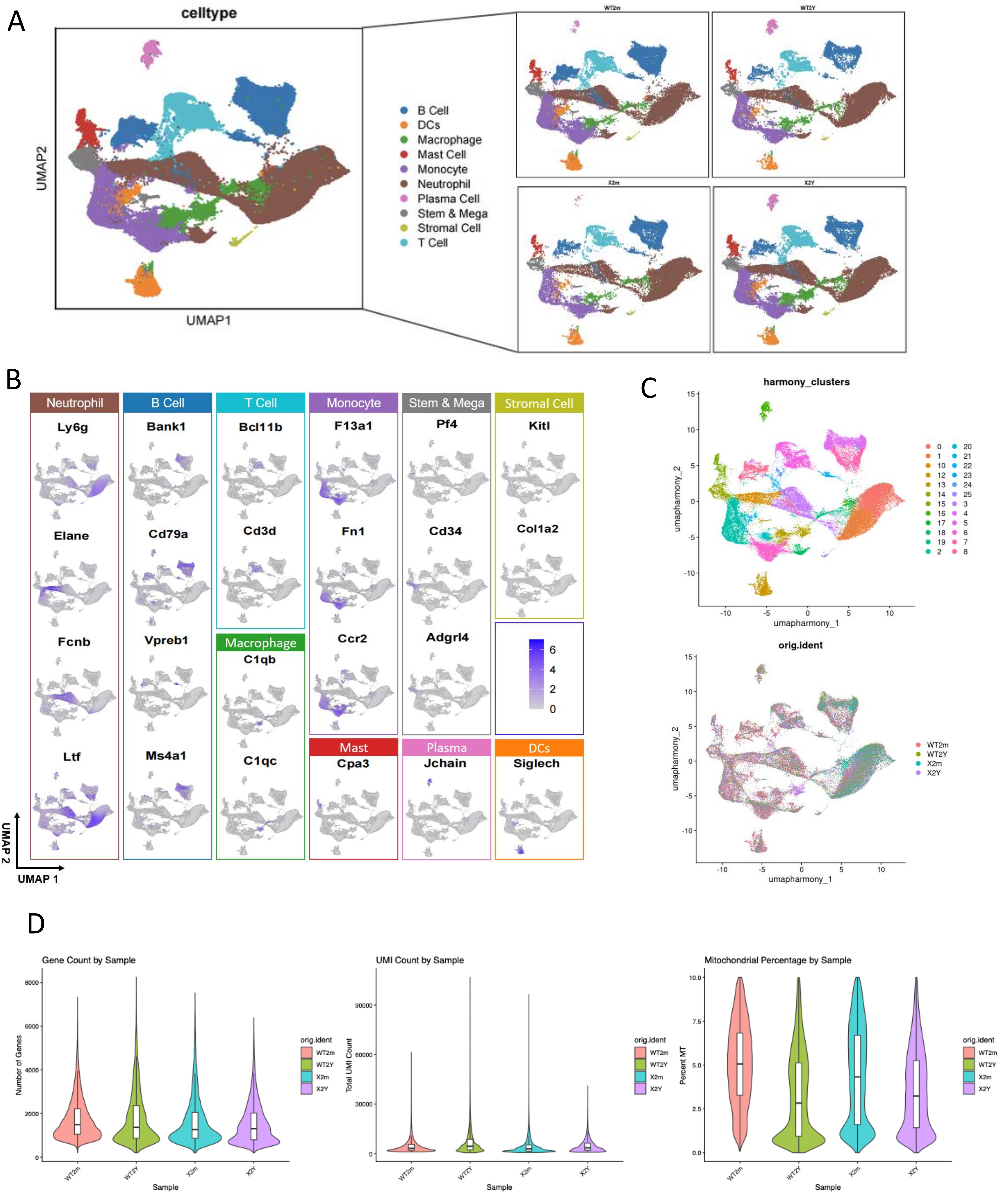

SDC Figure S1. Individual stages or features of the scRNA-Seq dataset analysis. (A) UMAP plots were generated for the four individual samples, revealing discernible differences between them. **(B)** Cell markers of different cell types (http://bio-bigdata.hrbmu.edu.cn/CellMarker). **(C)** UMAP of total samples with 24 clusters. **(D)** Quality control of scRNA-seq samples. (nFeature_RNA, nCount_RNA, percent.mt).

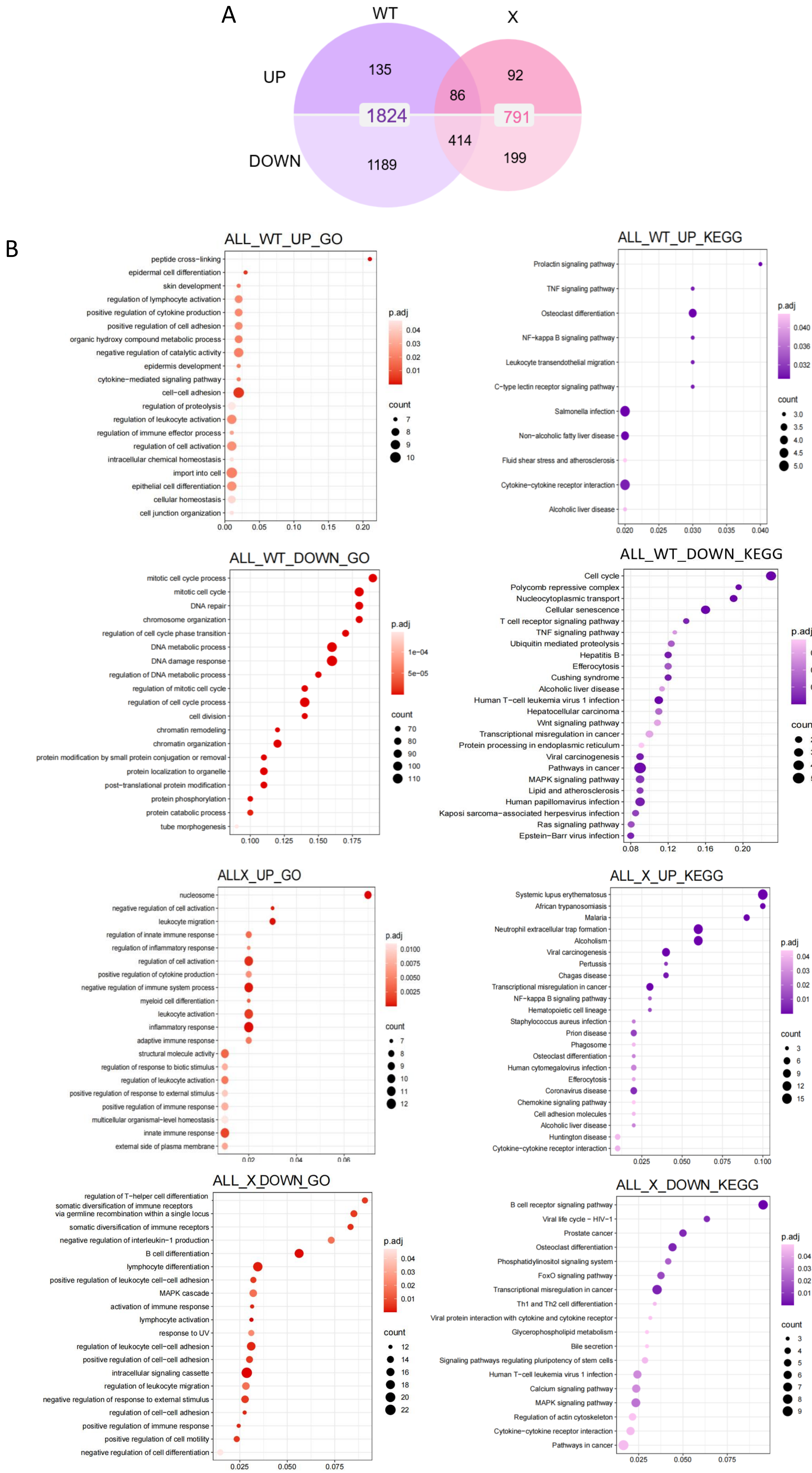

SDC Figure S2. Enrichment analysis of KEGG pathways and GO terms for genes differentially expressed after aging in WT and MXRA7-KO groups. (A) Venn plot of DEGs in WT and X groups. **(B)** KEGG and GO enrichment results.

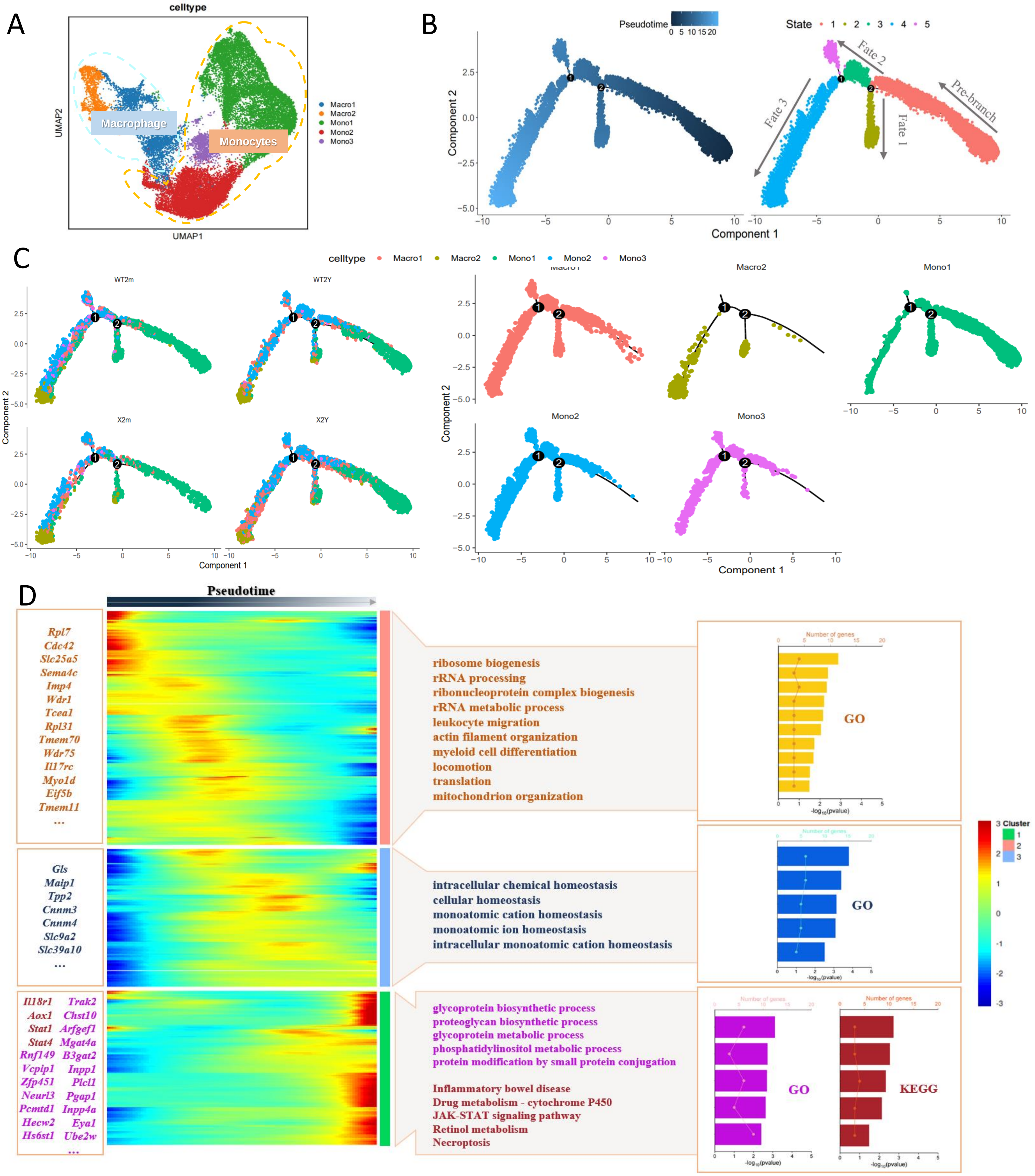

SDC Figure S3. Trajectory analysis reveals monocyte-macrophage differentiation and polarization dynamics. (A) Classification of monocytes (three subsets) and macrophages (two subtypes). **(B)** Pseudotime trajectory analysis of monocyte-macrophage differentiation. Left: Pseudotime trajectory plot (blue gradient: darker hues indicate initiation states; lighter hues denote terminal states). Right: Trajectory partitioned into three fates, with pink State 1 defined as the pre-branch differentiation origin (manually annotated). **(C)** Pseudotemporal dynamics by cell type. Left: Trajectory colored by sample group. Right: Cell type distribution across pseudotime: Mono1 dominates the pre-branch phase, while Mono2/Mono3 accumulate in mid-to-late phases. Macro1 persists throughout the trajectory, whereas Macro2 predominantly emerges in terminal phases, indicating late-stage differentiation. In terminal pseudotime (X2Y), Macro1 exhibits expanded proportions. **(D)** Gene dynamics and functional enrichment during differentiation. Top: Three gene clusters (pink State 1: early-upregulated; blue State 2: mid-upregulated; green State 3: late-upregulated) aligned with pseudotime progression. Bottom: Enriched pathways for each cluster (left: representative genes; middle: pathway terms; right: enrichment significance).

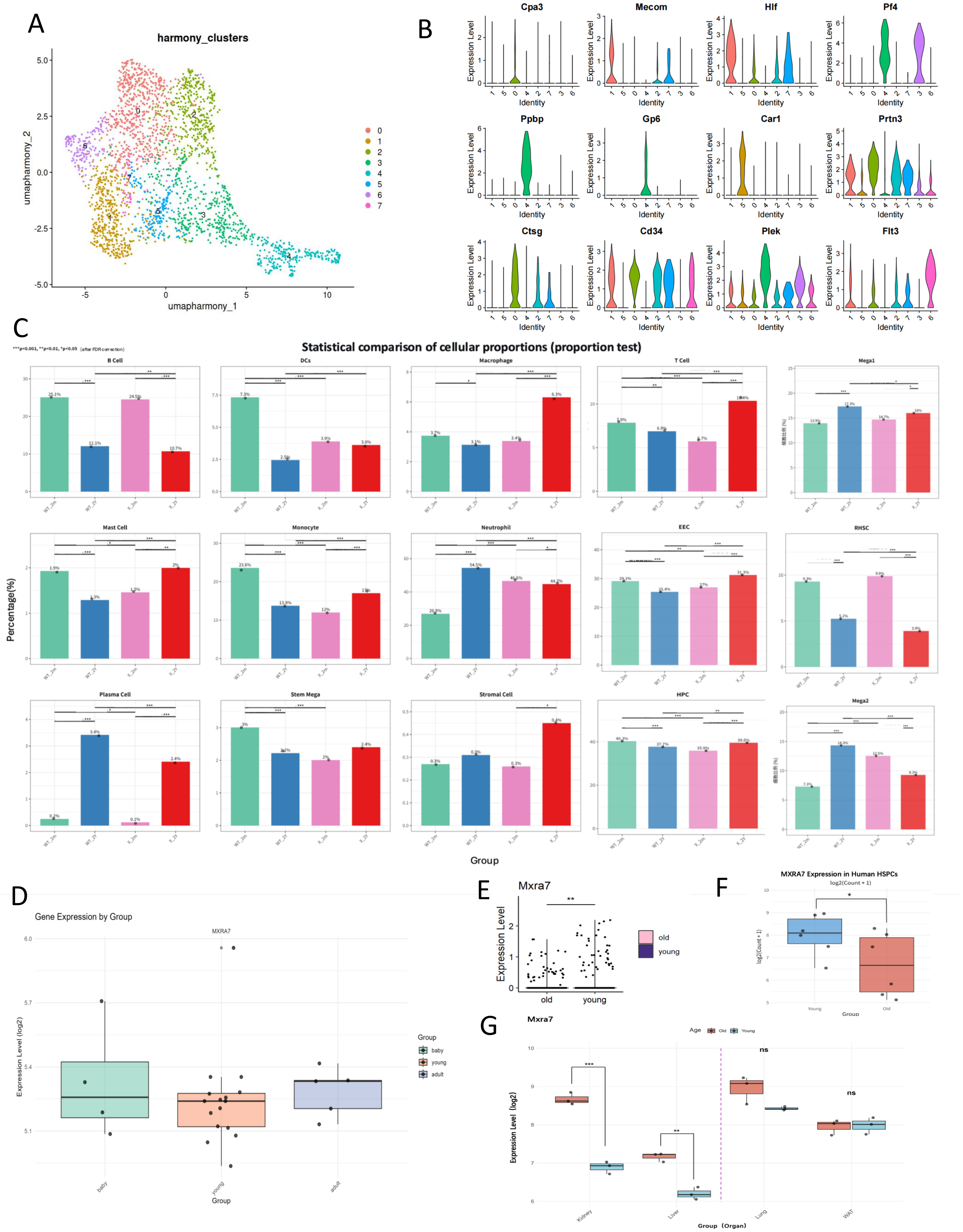

SDC Figure S4. MXRA7 expression in different cells or organs. (A) UMAP of HSCs & Megakaryocytes with 8 clusters. **(B)** Cell markers of different cell types (http://bio-bigdata.hrbmu.edu.cn/CellMarker; http://abc.sklehabc.com). **(C)** Statistical comparison of cellular proportions (proportion test). **(D)** The expression of *MXRA7* change dynamically by age in bone marrow (GSE11504). **(E)** In our own single-cell data, comparing only wild-type mouse bone marrow during aging, we found that *MXRA7* expression decreases with aging. **(F)** In human HSPC aging RNA-seq data (GSE243327), *MXRA7* expression was also found to decrease after aging**. (G)** In a mouse multi-organ aging dataset (GSE247440), *MXRA7* expression was found to increase in almost all organs after aging, although the changes in lung and white adipose tissue (WAT) were not statistically significant.

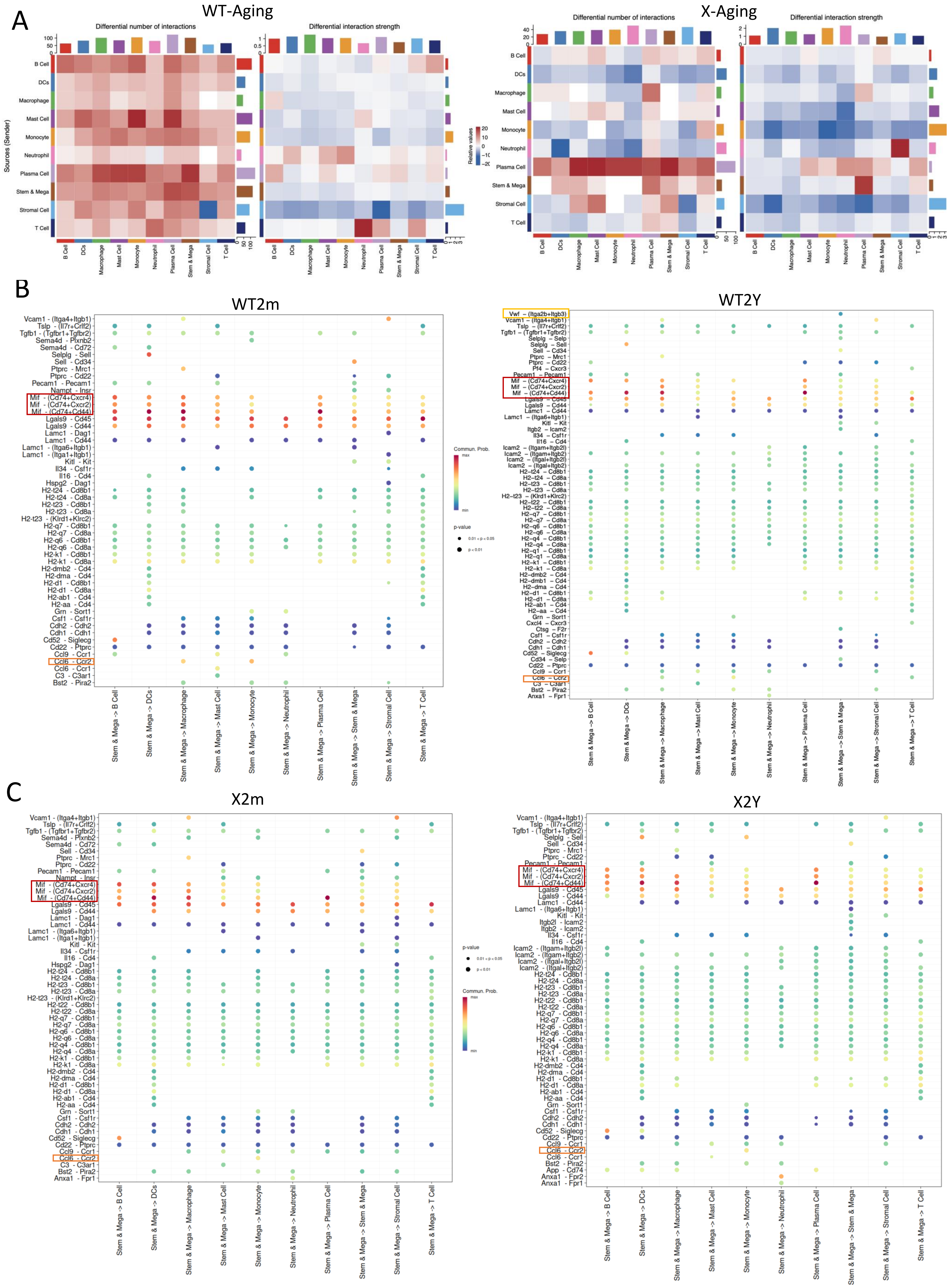

SDC Figure S5. Cell-Cell interactions under MXRA7 Deficiency. (A) Differential number and strength of interactions in both WT and *MXRA7*-KO groups. **(B-C)** Ligand-receptor pairs of cell–cell interactions in both WT and *MXRA7*-KO groups; the Mif (*Cd74+Cxcr4*) pair signal is lost in macrophage-hematopoietic cells(Stem & Mega) of aged *MXRA7*-KO mice; the *Ccl6+Ccr2* pair signal is absent in macrophage-hematopoietic cells (Stem & Mega) of both young and aged *MXRA7*-KO mice; and in aged wild-type mice, the Vwf (*Itga2b+Itgb3*) pair signal is lost in hematopoietic cells (Stem & Mega) following *MXRA7* deficiency.
